# Whole-Genome Sequences of Antibiotic-Resistant Aeromonas caviae Strains Isolated from Treated Wastewater

**DOI:** 10.1128/MRA.00645-20

**Published:** 2020-10-01

**Authors:** Łukasz Jałowiecki, Grażyna Płaza, Monika Nowrotek

**Affiliations:** a Environmental Microbiology Unit, Institute for Ecology of Industrial Areas, Katowice, Poland; Broad Institute

## Abstract

The presented data provide new information on antibiotic resistance and virulence genes in the genomes of Aeromonas caviae strains TW-2 and TW-6, isolated from treated wastewater. The results confirm the presence of multi-antibiotic-resistant Aeromonas caviae strains with virulence properties as “high-risk isolates” in treated wastewater.

## ANNOUNCEMENT

*Aeromonas* species are becoming food and waterborne pathogens, causing a wide spectrum of diseases in humans and animals (1, 2). Among the leading pathogenic species are Aeromonas hydrophila and Aeromonas caviae. They are emerging opportunistic human pathogens that cause skin and soft tissue infection and gastrointestinal tract infection, including hepatobiliary disease, diarrhea, and bacteremia (3–6).

In a preliminary study to screen for antibiotic-resistant bacteria, raw wastewater (RW) and treated wastewater (TW) samples were collected from a municipal wastewater treatment plant (WWTP) located in the southern part of Poland (50°5′35.881″N, 19°3′32.202″E). The isolation of antibiotic-resistant bacteria from wastewater and the antibiotic resistance and virulence phenotypes of *A. caviae* are described by Nowrotek et al. (7). The 16S rRNA sequences from strains TW-2 and TW-6 are deposited in GenBank under the accession numbers MN737498 and MN737502, respectively.

The genomic DNA of these strains was fragmented by sonication using a Covaris E210 instrument in accordance with the following parameters recommended for preparing libraries for sequencing with Illumina technology: duty cycle, 10%; intensity, 5; bursts per second, 200; treatment time, 100 seconds. Genomic libraries were constructed using the NEBNext DNA library prep master mix set for Illumina (New England BioLabs) according to the manufacturer’s recommendations. The libraries were then sequenced on the MiSeq sequencing platform (2 × 300-bp paired-end format; Illumina, Inc.) using the v3 Illumina kit according to the protocol “Preparing Libraries for Sequencing on the MiSeq” (15039740 rev. D).

The readings were filtered using the program Cutadapt v1.16. Sequences were assembled *de novo* using SPAdes v3.11.1 (8), and the NCBI Prokaryotic Genome Annotation Pipeline (PGAP) v4.12 was used for annotation.

Detection and determination of the antibiotic-resistance genes of these strains were performed using the ARIBA program v2.14.4 with database sequences from the Bacterial Antimicrobial Resistance Reference Gene Database (NCBI accession number PRJNA313047) (9). Virulence genes were detected using ARIBA v2.14.4 with database sequences from the VFDB database (10).

In summary, two genome sequences of Aeromonas caviae strains are presented. Our sequence data will aid in providing insights into the molecular mechanisms of antibiotic resistance and virulence. Our preliminary data suggest that the relation between the resistance phenotypes of some antibiotics from the β-lactam (amoxicillin, ampicillin, cefepime, cefradine, piperacillin, and ticarcillin) and aminoglycoside (tobramycin, neomycin, and vancomycin) groups and the presence of their genetic determinants in the tested strains might be observed (Table 1) (7). However, further and more accurate research is required to describe the concordance between the antimicrobial resistance genotypes and phenotypes; for example, the expression of the detected genes should be confirmed by the obtained results, and further comparative genomic analysis, such as analysis of the mobile elements, would elucidate the molecular mechanisms underlying the antibiotic resistance.

**TABLE 1:** Antibiotic resistance and virulence genes found in genomic DNA of Aeromonas caviae strains

*A. caviae* strain	Antibiotic resistance genes	Virulence genes
TW-2	*A7J11*; *aac(6)IIa*; *aac(6)Ib*; *aph(3)Ia*; *arr*; *bla*_MOX_; *bla*_OXA-10_; *bla*_OXA_38_; *catB3*; *mph_A*; *qacE*	*cheY*; *exeE*; *exeF*; *exeG*; *flhG*; *fliE*; *fliG*; *fliM*; *fliN*; *fliP*; *flmH*; *hlyA*; *mrkB*; *mrkC*; *mrkD*; *mrkF*; *pomA2*; *tapT*; *amoA*; *cheA*; *cheV*; *cheW*; *exeA*; *exeD*; *exeF*; *exeI*; *exeJ*; *exeN*; *flgC*; *flgL*; *flhA*; *flhB*; *fliA*; *fliF*; *fliI*; *fliL*
TW-6	*A7J11*; *mph_A*; *aac(6)Ib*; *aph(3)Ia*; *arr*; *qacE*; *bla*_OXA_10_; *bla*_OXA_38_; *catB3*; *bla*_MOX_; *aac(6)Iia*	*cheY_7*; *exeE*; *exeF*; *exeG*; *fliE_22*; *fliM_2*; *fliN_21*; *flmH*; *mrkB*^+^; *mrkD*^+^; *mrkF*^+^; *nueA_2*; *tapT*; *amoA*; *cheV_7*; *cheW_2_1*; *cluster_69*; *exeA_1*; *exeD*_*1*; *exeF*_*2*; *fleR*_*flrC*^+^; *flgC*_*18*; *flgG_10*; *flgK_16*; *flhA_17*; *flhB_31*; *flhB_32*; *flhB_38*; *flhG*^+^; *fliF_21*; *fliF_23*; *fliG_3*; *fliI_25*; *fliP_18*; *hlyA_4*; *hutA_4*; *mrkC^+^_1*; *pomA2*; *tapQ_1*; *tapU_1*

### Data availability.

The draft genome sequences of Aeromonas caviae isolates TW-2 and TW-6 have been deposited at DDBJ/ENA/GenBank under the accession numbers JABSNY000000000 and JABERS000000000 and the BioProject accession numbers PRJNA628854 and PRJNA629072, respectively. The raw reads for TW-2 and TW-6 are available in the Sequence Read Archive (SRA) under the accession numbers SRR11961868 and SRR11961867, respectively.
